# ChatGPT as a Virtual Patient: Written Empathic Expressions During Medical History Taking

**DOI:** 10.1007/s40670-025-02342-7

**Published:** 2025-02-27

**Authors:** Alexandra Aster, Sophia Viktoria Ragaller, Tobias Raupach, Ambra Marx

**Affiliations:** 1 Institute of Medical Education, University Hospital Bonn, Bonn, Germany; 2 Department of Psychosomatic Medicine and Psychotherapy, University Hospital Bonn, Bonn, Germany

**Keywords:** Empathy, ChatGPT, Medical education, History taking, Virtual patient

## Abstract

**Objective:**

Virtual patients are already utilized in the teaching of medical history taking. Since its emergence, ChatGPT has been integrated into several areas of medical education. This study aimed to examine whether ChatGPT can be used to train empathic history taking while fostering students’ subjective autonomy.

**Methods:**

Third-year medical students took histories with ChatGPT 3.5 after entering a predefined prompt covering cardiological diseases. Afterwards, students answered a questionnaire regarding their experienced autonomy. All chats were analyzed using the Empathic Communication Coding System measuring ChatGPT’s given empathic opportunities as well as students’ responses.

**Results:**

Out of 659 interactions, 93 were identified as empathic. ChatGPT provided opportunities mostly through reporting emotional statements or challenges. Students sometimes missed reacting adequately to ChatGPT’s opportunities but more often responded by implicit recognition of patient perspective and reported a high level of experienced autonomy.

**Conclusions:**

The study yielded preliminary results that ChatGPT might be suitable as a tool mimicking a virtual patient while enabling an empathic history taking. To date, ChatGPT seems valid as a supplement to training with simulated patients. Medical faculty could consider integrating ChatGPT into teaching, such as through a flipped classroom approach, to guide students in its use as ChatGPT continues to gain attention.

## Introduction

Empathy has not only been shown to improve the physician–patient-interaction, patients’ therapy adherence, and patient treatment outcomes but has also been shown to be a protective factor for physicians’ well-being [1–4]. Conversely, a physicians’ burnout negatively influences patients’ experience during a consultation [5]. Communication trainings, especially empathy interventions, significantly improve medical students’ empathy and have an impact on physicians showing more empathy in patient encounters afterwards [6, 7]. In this sense, the relevance of strengthening empathy during medical education and especially teaching empathic history taking becomes apparent. Generally, Cuff, Brown [8] contextualize empathy as follows:

“Empathy is an emotional response (affective), dependent upon the interaction between trait capacities and state influences. […] The resulting emotion is similar to one’s perception (directly experienced or imagined) and understanding (cognitive empathy) of the stimulus emotion, with recognition that the source of the emotion is not one’s own.” (p. 150)

During communication, empathy can be expressed threefold: through verbal, non-verbal, and paraverbal components. Non-verbal aspects are expressed through perceptible behaviors such as general posture and body movements [4, 9]. Paraverbal components are implicit aspects of communication that pertain to the expression of speech, such as vocal quality and prosodic characteristics [9]. Verbal aspects cover explicit as well as implicit reactions and responses to a message, for instance confirmation or support [10]. Another related factor in empathic communication is the technique of active listening as proposed by Rogers [11]. Active listening consists of three components: nonverbal engagement, verbal paraphrasing, and further questioning [12]. It has been shown to foster feelings of understanding and greater satisfaction [12] and is significantly associated with empathy in healthcare professions [13]. Beyond general empathy, the construct of clinical empathy that is defined as “a kind of emotional reasoning that allows physicians to incorporate emotional experiences as part of clinical decision-making” [14, p. 97] counts for the medical context. Clinical empathy has two sides of the same coin: it is emotional labor that healthcare professionals must manage effectively, but when routinized, it enhances medical encounters [15]. Researchers recommend incorporating practical communication training into medical education, emphasizing the hands-on aspect, as empathy must be developed through practice rather than theoretical instruction [4, 16]. Conducting communication trainings have been shown to help cardiologists improve their empathy [17]. To date, communication trainings in medical education are mostly conducted by using simulated patients (SP) [18]. According to Barrows [19], a simulated patient is a non-ill individual who, after thorough training, acts as a patient with a specified disease. Additionally, actual patients trained to present their own diseases in a standardized manner can also be classified under the umbrella term “standardized patients” along with simulated patients [19, 20]. However, virtual patients (VPs) have been increasingly and effectively used in recent years [21, 22]. Although SPs are widely recognized as a valuable component of communication training, implementing and maintaining an SP program is time-, labor-, and resource-intensive [23]. In contrast, while VPs do require resources to be set up, they offer learners the opportunity to repeat their training indefinitely [21] and shape their learning in a self-paced manner. Additionally, it is conceivable that using VPs may appeal to students’ feeling of autonomy, a basic psychological need according to the self-determination theory [24]. In a previous study, students favored the use of VPs compared to lectures when it comes to promoting self-directed learning [25]. Self-directed learning is linked with feelings of autonomy [26, 27], suggesting that students’ subjectively experienced autonomy is addressed by using VPs. In communication training, it is essential to develop not only hard skills, such as information gathering, but also soft skills, like empathy. Studies have shown that VPs can be effectively used to train empathic history taking [28, 29]. The advent of large language models, such as ChatGPT in November 2022, has created an opportunity to implement VPs. Literature suggests that for VPs to be effective in terms of learning outcomes, their textual setup must be based on valid theoretical foundations [21], which may also apply to the setup of ChatGPT as a VP. A handful of studies have utilized custom generative pre-trained transformers (GPTs), primarily based on ChatGPT, to train medical history taking in various fields, including dental [30] and medical education [31]. While SPs and VPs are already known to be feasible for practicing empathic history taking, it remains unknown whether ChatGPT can provide a feasible environment conducive to practicing empathic history taking that also supports students’ feelings of autonomy.

Therefore, within this study, the following research questions were assessed:

- RQ 1: Can ChatGPT be effectively used to conduct empathic history taking?
- RQ 2: How do students rate their experienced autonomy when taking a history with ChatGPT?

## Material and Methods

### Study Procedure

The study took place at a German medical school in summer term 2024 after being approved by the local ethics committee (application number: 2024–96-BO). Data collection directly followed each session of a course teaching the foundations of empathic history taking for third-year medical students. Each student participated in one session of the course. All attending students were invited to voluntarily participate in the study and were quasi-randomly assigned to one of four conditions. In the third year of their degree program, students begin their clinical training. Training in empathic communication is also provided through seminars and lectures in the semesters during the clinical training. Students provided implied informed consent by completing the study. Initially, they were instructed to take a written medical history with ChatGPT as a VP. However, they were not explicitly instructed to practice empathic communication, as this could have biased the results, for example due to social desirability. At this stage of their studies, students had not yet attended any courses covering clinical content. However, this is not a limitation, as the study aimed to assess empathic history taking, which was the primary learning objective of the course preceding the data collection. From a patient’s perspective, empathy is ranked among the top three most important factors in an emergency department [32]. Therefore, the storylines for the presented cases were based on cardiology and emergency medicine (Table 1). More specifically, endocarditis and heart failure were chosen as the two cardiological diseases for the study. Additionally, it was of interest whether a change in the temperature of ChatGPT’s response has an impact on students’ expressed empathy. Specifying the temperature adjusts ChatGPT’s responses in terms of creativity by influencing how likely words are strung together. In this sense, a lower temperature (e.g., 0.2) yields more focused and concrete answers, as subsequent words are chosen based on higher probabilities [33, 34]. Conversely, a higher temperature (e.g., 0.8) results in more random and creative responses by selecting words based on lower probabilities, making it more suitable for training communicative skills [33, 34]. Both diseases were presented with a response temperature of either 0.2 or 0.8, resulting in the four conditions: endocarditis 0.2, endocarditis 0.8, heart failure 0.2, and heart failure 0.8. The respective prompt was given to students via an online storage platform. Students pasted the prompt in their own ChatGPT 3.5 accounts and had 60 min to perform the history taking. Throughout and after the session, no feedback was provided to the students regarding either the content of the medical histories or their expressed empathy. Students anonymously shared their chat transcripts for data analysis by providing the export link for the respective chat and completed the short questionnaire described below.

**Table 1:** Structure of applied ChatGPT prompt

Structure	Prompt elements
Basic structure part 1	Hello. Assume the role of a standardized patient, adjust your responses according to the patient's condition, and independently decide the level of detail in your answers. A standardized patient is an actor who portrays the role of a real patient as authentically as possible. Therefore, the standardized patient does not understand or interpret the clinical findings provided below and cannot answer any regarding questions. Do not use medical jargon in your responses. Response temperature: 0.2 / 0.8
Medical information 1 (endocarditis)	An 81-year-old man presents to the emergency department, reporting fatigue. He arrived at the emergency department on his own and has a reddish face color. Additionally, he reports suffering from muscle pain. Osler nodes and positive blood cultures are observed
Medical information 2 (heart failure)	A 75-year-old man presents to the emergency department reporting shortness of breath. He was brought in by emergency services and appears pale. Additionally, he reports frequent evening ankle swelling. Initial physical examination reveals the following clinical findings: the first heart sound is relatively quiet, a holosystolic murmur at the 5th intercostal space, left midclavicular line, and a slightly elevated jugular venous pressure
Basic structure part 2	You will now be asked medical history questions, which you should answer accurately in your role as a standardized patient, according to the case. The illness itself must not be mentioned at any point during the medical history. The history-taking process ends only when the correct diagnosis is made. Introduce yourself with a full name at the beginning of the history-taking. Do not ask any questions at the start

A single response temperature was chosen for each condition

### Materials

#### Development of the ChatGPT Prompt

To enable ChatGPT to act as a VP, a basic structure instructing ChatGPT how to take on its role was created for each prompt, which was then tailored with the specific story of the respective disease. Table 1 shows the prompt structure, which — for the purpose of this publication — was translated to English since the study was conducted in German. For each condition, a disease storyline and a response temperature were selected. To ensure external validity, the disease was not explicitly named in the prompt, as most patients do not present with a final diagnosis in real life. The basic structure part 2 included instructions that history taking should continue until the correct diagnosis was made. However, entering a diagnosis was not mandatory, and students could conclude history taking whenever they felt they had gathered sufficient information. However, it has proven to be effective during the preceding prompt testing to add this instruction as a security measure for ChatGPT to stay in its role and to not accidentally name the disease.

#### Questionnaire

A short questionnaire was developed to measure two variables. The first variable assessed students’ self-reported feelings of autonomy during the history taking with ChatGPT, using two autonomy scales—i.e., freedom of choice, and task relevance—postulated by Sailer [35]. Both scales were adapted to fit the specific context of history taking (e.g., “I was able to decide for myself, which history taking questions I wanted to ask.”). All six questions were measured using a 7-point Likert scale. The second variable assessed students’ prior use of ChatGPT, ranging from no experience to extensive experience on a 5-point scale.

### Choice of Tool for Data Analysis

To assess empathic interactions, the Empathic Communication Coding System (ECCS) [10, 36] was applied, as it allowed for separately coding ChatGPT’s provided empathic opportunities and medical students’ corresponding empathic responses. ChatGPT’s statements were analyzed as opportunities according to the ECCS. According to the manuals provided by the International Association for Communication in Healthcare [EACH, 37], opportunities can be provided on three different levels while responses can be provided on seven levels. For an overview and a description of the levels, see Table 2.

**Table 2:** Overview and descriptions of the levels used for coding ChatGPT’s opportunities and students responses

ChatGPT’s opportunities		Student responses	
Level	Description	Level	Description
1	*Emotion statements* ChatGPT describing current feelings of emotions	0	*Denial of ChatGPT’s perspective* Ignoring or disconfirming ChatGPT’s perspective
2	*Progress statements* ChatGPT describing positive developments or an improved quality of life	1	*Perfunctory recognition of ChatGPT’s perspective* Superficial recognitions of ChatGPT’s perspective without explicitly acknowledging it (e.g. “hmm”). Hence, this level cannot be adequately represented in exclusively written communication with ChatGPT
3	*Challenge statements* ChatGPT describing the negative impact that problems have on the quality of life or reports of radical life events	2	*Implicit recognition of ChatGPT’s perspective* Implicit recognition without focusing on the central issue but pointing out a peripheral aspect
		3	*Acknowledgement without pursuit* Acknowledging ChatGPT’s statements without further pursuit
		4	*Acknowledgement with pursuit* Acknowledging ChatGPT’s statements with further pursuit
		5	*Confirmation* Confirmation of ChatGPT’s perspective that can be expressed through various ways
		6	*Shared Feeling or Experience* Sharing their own feelings or experiences with ChatGPT

Based on the works by Bylund and Makoul [10] and Bylund and Makoul [36], and the manuals provided by EACH [37]. All headings are extracted exactly from the manuals, with the exception of the word patient being changed to ChatGPT

Coding ChatGPT’s provided opportunities enabled verification of whether it had offered empathic opportunities at all. For ChatGPT to be effectively used as a VP for training empathic communication, it must independently generate empathic opportunities, as these cannot be as easily preconfigured in the publicly available version as in predefined VPs. Thus, it is essential not only to analyze students’ written empathic responses but also to evaluate ChatGPT’s suitability as a VP in imitating human conversation and interaction. This approach enables the examination of empathic interplay and the analysis of interactions between medical students and ChatGPT as a VP.

### Data Analysis

Two psychologists independently rated each student’s history taking chat protocol using the ECCS. Beforehand, both raters became acquainted with the coding scheme by studying the manuals provided by EACH [37]. During the rating, the raters adhered to the manuals and referred to it in case of ambiguous ratings. Additionally, three test ratings with chat protocols, which were not included in the final analysis, were conducted to ensure both raters reached a common understanding. In contrast to a face-to-face conversation, interaction with ChatGPT does not constitute a traditional dialogue, as ChatGPT provides a response to which the user reacts in isolation. Consequently, both raters evaluated each pair comprising ChatGPT’s statement and the subsequent medical students’ reaction. If ChatGPT did not present an empathic opportunity, it was coded as “not applicable”. Students’ responses were also coded as “not applicable” due to the absence of an opportunity for the student to react empathetically. Interrater reliability was assessed using the intraclass correlation coefficient, resulting in a good agreement of 0.770, according to Cicchetti [38]. Both raters resolved discrepant ratings, resulting in agreed-upon values for each rating used for the analysis.

## Results

Thirty-five third-year medical students participated in the study, with a total of 28 valid chat protocols included. The discrepancy between the number of participants and valid chat protocols was due to technical issues with exporting the chat data. No additional exclusion criteria for chat protocols were applied. On average, students made 24.96 (*SD* = 9.41) entries in ChatGPT during their history takings.

### Empathy Ratings

Out of 659 general interactions (i.e., all students’ entries and ChatGPT statements taken together irrespective of empathic content), 93 (14%) empathic interactions between ChatGPT and medical students could be identified across both diseases and response temperatures. The highest number of empathic interactions was observed for endocarditis, with 27 interactions at both response temperatures. All empathic opportunities and responses broken down to each disease and the respective response temperature can be found in Table 3. A detailed analysis of both interaction components, ChatGPT’s empathic opportunities and students’ responses, is presented in the following paragraphs.

**Table 3:** Descriptive distribution of levels in opportunities and responses

Levels of the respective opportunities and responses			
**All diseases and response temperatures** (*N* = 28)			
*ChatGPT’s opportunities*		*Student responses*	
Level 1 = 50 Level 2 = 2 Level 3 = 41		Level 0 = 34 Level 1 = 0 Level 2 = 41 Level 3 = 3 Level 4 = 7 Level 5 = 8 Level 6 = 0	
**Response temperature 0.2 across both diseases** (*n* = 16)		**Response temperature 0.8 across both diseases** (*n* = 12)	
*Opportunity*	*Response*	*Opportunity*	*Response*
Level 1 = 28 Level 2 = 2 Level 3 = 22	Level 0 = 18 Level 1 = 0 Level 2 = 20 Level 3 = 2 Level 4 = 6 Level 5 = 6 Level 6 = 0	Level 1 = 22 Level 2 = 0 Level 3 = 19	Level 0 = 16 Level 1 = 0 Level 2 = 21 Level 3 = 1 Level 4 = 1 Level 5 = 2 Level 6 = 0

The levels arranged parallel do not match correspondingly to each other but independently represent the respective numbers

ChatGPT primarily provided empathic opportunities at level 1 (*n* = 50) across all conditions, directly followed by level 3 (*n* = 41) and level 2 (*n* = 2). The number of provided opportunities did not differ significantly between response temperatures (*U* = 1045.00, *p* = 0.852). ChatGPT predominantly used statements of emotion or challenge, while progress statements were rarely expressed. It maintained its role stringently and coherently. Descriptively, ChatGPT provided slightly more emotion-related statements at the lower response temperature, where only two progress statements were detected. A closer look at the questions posed by students revealed that ChatGPT allowed students to ask a substantial number of relevant questions and provided satisfactory answers. From a qualitative perspective, it is striking that ChatGPT commonly draws on the same storylines, expanding beyond the information provided in the prompt, particularly within the same session. Moreover, it is evident that ChatGPT adjusts its responses based on the students’ questioning behavior.

**Fig. 1:**
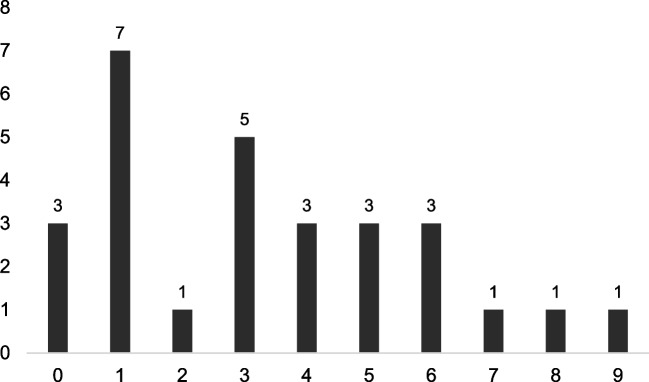
Number of empathic responses per chat and distribution across all chats. X-axis, number of empathic responses per chat; Y-axis, number of occurrence of the respective number across all chats, which is also depicted above each bar

Students’ mostly showed empathic responses at level 2 (*n* = 41) across all diseases and response temperatures. The number of empathic responses did not differ significantly between the response temperatures (*U* = 912.00, *p* = 0.200). Although only a descriptive trend, slightly more responses were observed at levels 5 and 6 for the lower response temperature. Across all chats, students most commonly provided either one or three empathic responses per chat (Fig. 1). However, in 34 instances, students did not respond to ChatGPT’s empathic opportunities at all.

**Fig. 2:**
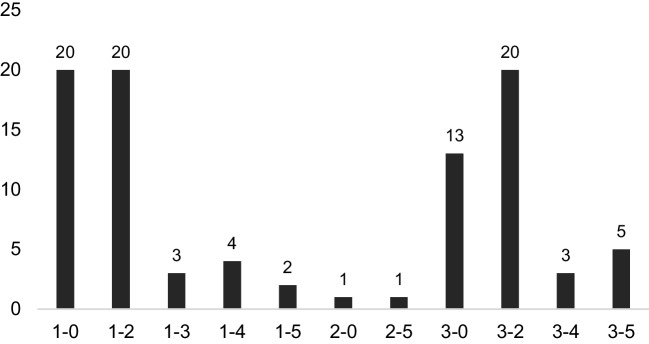
Distribution and absolute number of opportunity-response pairs across all diseases and response temperatures. X-axis, opportunity-response pairs with the left number representing the level of the opportunity and the right number representing the level of the response; Y-axis, absolute number of occurrences, which is also depicted above each bar

Specifically, the most frequent empathic interactions occurred with the level combinations 1–0, 1–2, and 3–2 with 20 occurrences each, irrespective of disease and response temperature. Figure 2 illustrates the distribution as well as the different levels on which opportunity-response pairs occurred.

### Students’ Feelings of Autonomy

Twenty-five students completed the questionnaire regarding perceived autonomy. When considering both scales together, students reached an average of *M* = 38.2 (*SD* = 3.44) out of a maximum of 42. Broken down to the single scales, students rated their autonomy on the scale “freedom of choice” with an average of *M* = 6.8 (*SD* = 0.54) out of a maximum of 7. On the scale “task relevance”, students rated their experienced autonomy with an average of *M* = 5.93 (*SD* = 1.09) out of a maximum of 7. The study in which the scale was developed [35] can be referenced to obtain mean values and standard deviations for comparing with autonomy scores in the current study. In the referenced study, the gamification group had an average of *M* = 4.03 (*SD* = 1.49) on the “freedom of choice” subscale and *M* = 5.46 (*SD* = 1.06) on the “task relevance” subscale [35]. In comparison, subjective autonomy when using ChatGPT was higher in the current study.

In a separate question, students rated their previous experience with ChatGPT. It showed that students’ experience was nearly normally distributed with the majority of students reporting a medium level of previous experience (*M* = 2.48, *SD* = 0.98).

## Discussion and Conclusion

To our knowledge, this is the first study to examine whether medical students can practice empathic history taking with ChatGPT as a virtual patient. Given ChatGPT’s widespread recognition and accessibility, assessing its effectiveness for medical training is essential. ChatGPT’s empathy has been examined in the context of responding to patient questions [39], and patients’ perceptions of ChatGPT’s responses compared to specialists’ responses [40], but not yet within the context of practicing empathic history taking. The study results indicated that while ChatGPT was able to provide empathic opportunities, the majority of its statements were factual rather than empathic. Building on this, students were able to show empathic responses, although the responses were still predominantly factual. Nevertheless, a relatively small number of empathic interactions between students and ChatGPT occurred. Taken together, ChatGPT in its free version presents certain challenges but may also serve as a feasible tool for practicing empathic communication. Adjusting the response temperature in the prompt did not result in any significant differences in the number of provided opportunities or in the responses given. Moreover, students reported high feelings of autonomy during history taking. Collectively, the results suggest that ChatGPT might be a potentially valuable and feasible option for practicing history taking, even though the results can only be interpreted as preliminary at this stage and further research is needed.

Given that students demonstrated adequate questions on a medical level, it is worth questioning whether ChatGPT might be more suitable for learning the content of history taking rather than training empathic communication. Supporting this assumption, Deladisma and Cohen [41] demonstrated that medical students interacted empathically with VPs, but interactions were insufficient on a quantitative and qualitative level to replace real-life interactions such as encounters with SPs. At the end of the session, some students verbally reported feeling hindered in expressing empathy due to the nature of ChatGPT requiring questions to be entered in writing into a technical device. Others reported difficulty in demonstrating empathy towards an AI as a communication partner. However, since ChatGPT provided humanoid conversation, the technical aspect might be a more valid reason than the quality of communication. These assumptions should be validated in future studies by systematically assessing students’ perception of their learning experience when using ChatGPT. In the future, there will be further developments of ChatGPT (e.g., communicating via vocal or visual input) that might facilitate showing empathy through imitating an even more humanoid conversation possibly including non-verbal communication aspects. In addition to the forthcoming updates to ChatGPT, previous research has attempted to integrate ChatGPT or other large language models into interfaces designed to facilitate empathic, dialog-based interactions [42]. One advantage is the inclusion of an integrated feedback system [42], which, in our case, had to be manually added into the prompt. Although only a few empathic interactions were observed, students conducted sufficient history takings, supporting the assumption that ChatGPT is not yet unconditionally applicable as a tool for practicing empathic history taking, but can be useful for general history taking. However, it is plausible that embedding the desired ECCS levels directly into the prompt could encourage ChatGPT to provide empathic opportunities aligned with these levels. Furthermore, it is conceivable that specifying these levels, along with a corresponding request, could prompt ChatGPT to provide targeted feedback on students’ responses. Nevertheless, ChatGPT might be an adjunct to learning with SPs. Future studies should compare the effectiveness of training empathic history taking using ChatGPT with SP-based training. Communication generally consists of verbal and non-verbal aspects and it has already been shown that empathy is correlated with non-verbal communication in medical encounters but not with verbal communication [4]. An obstacle to consider when using ChatGPT for training history taking is that non-verbal aspects cannot yet be addressed by using ChatGPT. The same applies to minimal implicit verbal expressions (e.g., “hmm”), as these are inserted spontaneously during a conversation that cannot be effectively replicated in interactions with ChatGPT. The study results accentuate this assumption as no empathic responses at level 1 were found. On the other side, there were also no findings for responses at level 6. It should be discussed whether responding at level 6 with shared feelings or expressions constitutes too much self-disclosure, and whether the optimal empathic response might be found closer to the middle of the spectrum with balanced and personalized self-disclosure. A study conducted with patients suffering from chronic pain found that self-disclosure is conducive to patients’ own self-disclosure and is perceived as empathic; however, patients also reported concerns that excessive self-disclosure by the physician might lead to an insufficient focus on the patient [43]. Nevertheless, self-disclosure is a connective element in a physician–patient relationship as physicians sharing appropriate self-disclosure are perceived as empathic and facilitate patients’ self-disclosure [44]. Since expressing empathy is highly dependent on culture [45], the results may differ across different regions, and, therefore, may not be fully generalizable.

### Strengths and Limitations

To our knowledge, this is the first study using ChatGPT as a virtual patient presenting with cardiological diseases in an emergency ward. Without a doubt, empathy plays an important role in the practice of medicine and is stated as crucial for patient satisfaction in the areas of cardiology and emergency medicine [32], which is why we combined both areas. During the analysis, both sides of the interaction between ChatGPT and medical students were rated, resulting in an assessment of ChatGPT’s given empathic opportunities and students’ empathic reactions. Two main conclusions can be drawn. The first concerns whether ChatGPT can be used for practicing empathic communication from the students’ perspective. The second relates to ChatGPT’s feasibility in creating an appropriate environment for such practice by providing empathic opportunities. In this context, it is noteworthy that students conducted an average of 24.96 interactions per chat, demonstrating ChatGPT’s ability to maintain its persona over an extended conversation. Moreover, the use of their own ChatGPT accounts by students was advantageous, as it enhanced the external validity by providing insight into how students might use ChatGPT as a VP at home. Although most students reported a moderate experience with ChatGPT, its ever-increasing usage worldwide suggests that an increasing number of medical students will likely use ChatGPT at home.

The study has limitations, the most significant being the small sample size. As a result, the findings should be considered preliminary. Future research should include larger and more diverse samples. One shortcoming that simultaneously is an advantage is the use of students’ own ChatGPT accounts. For this study, no separate interface was created; and therefore, students had to use their own accounts. While this improved external validity, it reduced controllability. One obstacle was that students should not see the name of the disease in the applied prompt, which is why the disease had to be embedded in the storyline paraphrased by only stating relevant facts. This obstacle could be circumvented by creating an interface in which the disease could be deposited namely along with all specifications [e.g., 31]. The study results showed no significant differences in verbal empathy expressions between the two temperatures. Therefore, it should be noted that simply adding information about response temperature to the prompt is insufficient. A customized interface would also allow for clearly depositing the response temperature on which ChatGPT should provide its answers. Another limiting factor is that ChatGPT 3.5 is not as controllable as a customized VP, especially in terms of the storyline. During the data collection, ChatGPT occasionally provided trial versions of ChatGPT 4.0 for the first questions in some chats, which was not controllable. The course students attended beforehand focused on general history taking rather than disease-specific history taking. Although empathy is important in the field of cardiology and emergency medicine, applying the chosen diseases simultaneously is a limitation. Future studies should match the diseases with existing course contents. Nevertheless, students were able to transfer their learned skills to new contexts favoring the generalizability of the learned contents. Moreover, the emergency department as a chosen setting for the storyline in the prompt may have hindered students from taking an empathic medical history. Although the selected diseases were not life-threatening, the study should be conducted in general practice setting, where such conditions might be approached more empathically due to their less severe nature. The ECCS is a standardized and valid tool for analyzing empathic communication; however, until now, it has only been used in research involving human participants. Therefore, it should be further investigated whether the ECCS is also a valid tool for analyzing a conversation between a human and a large language model. For instance, empathic opportunities at level 1 could not be coded since this level refers to expressions that are shown within a human conversation but not in a conversation with a large language model.

### Conclusion

The study showed initial results for ChatGPT being used as a tool for practicing empathic history taking. Although only a relatively small number of empathic interactions were identified among the many interactions between ChatGPT and medical students, students were still able to take comprehensive histories. Thus, ChatGPT might not yet be perfectly suited for practicing empathic history taking, but it might be effective for training comprehensive history taking. Future research should replicate the study with a larger sample size to draw more robust and reliable conclusions that extend beyond the preliminary findings. Conclusively, the free version of ChatGPT may serve as a useful and cost-effective supplement to SPs for factual learning and, initially, for empathic communication training.

### Practice Implications

Using ChatGPT might be a feasible addition for practicing empathic history taking. ChatGPT is a freely accessible tool making it possible for students to practice their history taking skills self-paced and indefinitely in their everyday lives. Teachers need to moderate to ensure an effective learning environment, for example, by providing validated prompts. Taking this idea further, training history taking with ChatGPT might be implemented in a flipped-classroom model. In this sense, students prepare themselves for the course by taking a history with a predefined prompt asynchronously before the course itself. During the synchronous part of the course, the prepared chats are reviewed and teachers should teach the cognitive aspects of empathy during history taking. In the following asynchronous part, ChatGPT can again be used as a tool for practicing the learned aspects of empathic history taking. In a summative exam (e.g., OSCE), the effectiveness of ChatGPT as a training tool could be assessed based on performance improvement. Additionally, training with ChatGPT should be compared to learning with simulated patients.

## Data Availability

The datasets used and/or analyzed during the current study are available from the corresponding author on reasonable request.
